# In Vitro Enzyme Kinetics and NMR-Based Product Elucidation for Glutathione S-Conjugation of the Anticancer Unsymmetrical Bisacridine C-2028 in Liver Microsomes and Cytosol: Major Role of Glutathione S-Transferase M1-1 Isoenzyme

**DOI:** 10.3390/molecules28196812

**Published:** 2023-09-26

**Authors:** Agnieszka Potęga, Dominika Rafalska, Dawid Kazimierczyk, Michał Kosno, Aleksandra Pawłowicz, Witold Andrałojć, Ewa Paluszkiewicz, Tomasz Laskowski

**Affiliations:** 1 Department of Pharmaceutical Technology and Biochemistry, Faculty of Chemistry, Gdańsk University of Technology, Gabriela Narutowicza Str. 11/12, 80-233 Gdańsk, Poland; s177471@student.pg.edu.pl (D.R.); s166007@student.pg.edu.pl (D.K.); michal.kosno@pg.edu.pl (M.K.); ewa.paluszkiewicz@pg.edu.pl (E.P.); tomasz.laskowski@pg.edu.pl (T.L.); 2 Institute of Bioorganic Chemistry, Polish Academy of Sciences, Zygmunta Noskowskiego Str. 12/14, 61-704 Poznań, Poland; apawlowicz@ibch.poznan.pl (A.P.); wandralojc@ibch.poznan.pl (W.A.)

**Keywords:** anticancer unsymmetrical bisacridine, glutathione S-conjugate, GSTM, enzyme kinetics, NMR-based product elucidation

## Abstract

This work is the next step in studying the interplay between C-2028 (anticancer-active unsymmetrical bisacridine developed in our group) and the glutathione S-transferase/glutathione (GST/GSH) system. Here, we analyzed the concentration- and pH-dependent GSH conjugation of C-2028 in rat liver microsomes and cytosol. We also applied three recombinant human GST isoenzymes, which altered expression was found in various tumors. The formation of GSH S-conjugate of C-2028 in liver subfractions followed Michaelis-Menten kinetics. We found that C-2028 was conjugated with GSH preferentially by GSTM1-1, revealing a sigmoidal kinetic model. Using a colorimetric assay (MTT test), we initially assessed the cellular GST/GSH-dependent biotransformation of C-2028 in relation to cytotoxicity against Du-145 human prostate cancer cells in the presence or absence of the modulator of GSH biosynthesis. Pretreatment of cells with buthionine sulfoximine resulted in a cytotoxicity decrease, suggesting a possible GSH-mediated bioactivation process. Altogether, our results confirmed the importance of GSH conjugation in C-2028 metabolism, which humans must consider when planning a treatment strategy. Finally, nuclear magnetic resonance spectroscopy elucidated the structure of the GSH-derived product of C-2028. Hence, synthesizing the compound standard necessary for further advanced biological and bioanalytical investigations will be achievable.

## 1. Introduction

**Figure 1:**
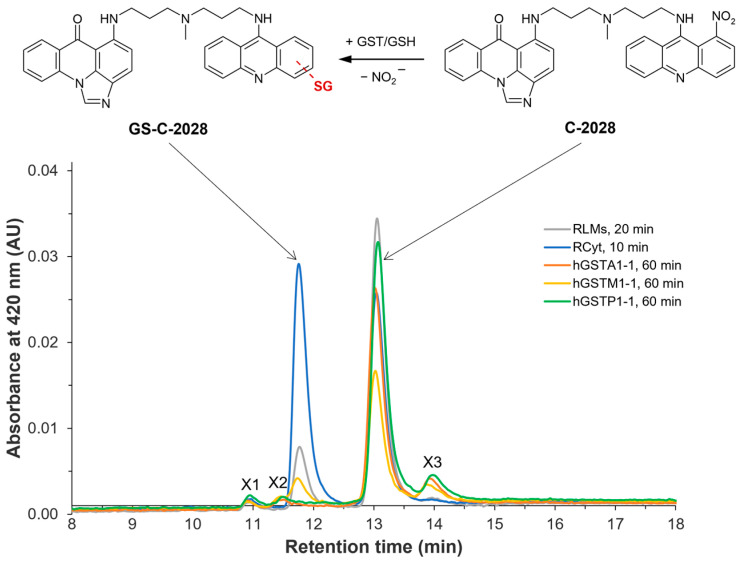
Representative HPLC chromatograms of unsymmetrical bisacridine C-2028 and/or its GSH S-conjugate (GS-C-2028) in rat liver microsomes (RLMs) and cytosol (RCyt) as well as with recombinant human GSTs (A1-1, M1-1, and P1-1) after the indicated incubation time. For experimental details, see the Materials and Methods section (Section 4). The scheme of possible GST/GSH-mediated conjugation reaction of C-2028 was shown at the top [8].

C-2028 (9′-{N-[(imidazo [4,5,1-de]-acridin-6-on-5-yl)aminopropyl]-N-methylaminopropylamino}-1′-nitroacridine), selected for the current investigations (a structure presented in Figure 1 at the right top), is a promising anticancer drug candidate from a novel patented class of unsymmetrical bisacridine (UA) derivatives synthesized and developed in our laboratory [1]. Several data demonstrated that C-2028 is highly active against various cancer cell lines, Walker 256 adenocarcinoma in rats, and human tumor xenografts in nude mice, including pancreatic, colorectal, and lung cancers [1]. Further studies on the biological effect of this compound revealed its ability to inhibit the growth and viability of 3D spheroids derived from cancer cells, which are a promising tool in drug development and testing [2]. Additionally, previous results established that cancer cells treated with C-2028 undergo apoptosis or senescence [3]. It was also found that conjugation of the C-2028 molecule with the quantum dots−*β*−cyclodextrin−folic acid (QD−*β*−CD−FA) vehicle significantly improved cellular uptake and drug release from nanoconjugate in cancer cells. Notably, C-2028 concentration was increased in cell organelles characterized by low pH, such as lysosomes and endosomes [4]. A detailed physicochemical characterization of this compound gave insight into its molecular properties in aqueous media, including protonation state, self-association ratio, and solubility [5]. Recent experiments showed that C-2028 and other UAs exhibited well-defined interactions with several DNA G-quadruplexes, which are currently regarded as very attractive molecular targets in anticancer therapy [1]. This supported previous reports on the ability of UAs to inhibit the expression of K-Ras in Panc-1 pancreatic cancer cells [1] and c-Myc in HCT116 colorectal and H460 lung cancer cells [6]. The knowledge about the molecular background of UA action is constantly increasing. Nonetheless, to predict the behavior of the drug in the patient’s body, its metabolism studies with phase I and phase II drug-metabolizing enzymes are also necessary.

Initial investigations showed that under in vitro conditions, C-2028 underwent metabolic transformations in noncellular systems (i.e., in the presence of human and rat liver microsomal enzymes) and cancer cells [7]. We found that the main transformation pathway of the compound was the nitro group reduction with cytochrome P450 (P450) isoenzymes 3A4 and 2C19, as well as metabolism to N-oxide derivative with flavin-containing monooxygenase 1 (FMO1). Most metabolites of C-2028 retained a dimeric structure. Moreover, they were generally consistent with the products generated electrochemically [7]. Concerning phase II metabolic reactions and the involvement of conjugative enzymes, no glucuronidation of C-2028 was observed, but the compound modulated the activity of UDP-glucuronosyltransferase (UGT) isoenzymes 1A and 2B [7].

Further, we showed that C-2028 was rapidly conjugated with glutathione (GSH) to only one main product (GSH S-conjugate) via the catalytic action of rat microsomal and cytosolic GSH S-transferases (GSTs) [8]. No kinetic data for this reaction have been assessed yet. It should be emphasized that we excluded prior cytochrome P450-mediated activation of the parent compound to form GSH S-conjugate. Additionally, we also reported the non-enzymatic GSH-mediated metabolic pathway of C-2028. Finally, mass spectrometry data analysis indicated that the modifications of the C-2028 molecule are likely located in the acridine ring system containing the nitro group (a proposed structure presented in Figure 1 at the left top).

The above findings suggest the important role of the GST/GSH system in the metabolism of UA derivatives. The co-action of GST isoenzymes and GSH is generally recognized as a significant detoxifying process of numerous endogenous and exogenous compounds, including anticancer drugs [9,10]. However, enhanced drug detoxification, often associated with GST overexpression in many types of cancers, is used by cancer cells to develop drug resistance and improve their survival [11]. On the other hand, the conjugation of the drug with GSH can occasionally result in bioactivation reactions as it can generate harmful reactive conjugates, as is the case, e.g., with the anticancer busulfan and sulofenur [10,12,13]. Thus, GST/GSH conjugation testing is important to determine the therapeutic efficacy of a drug and/or to predict its potential toxicity. Research in this area is currently of great interest, as their results provide the background for the rational design of individual and multidrug anticancer therapies. Our previous findings that the most anticancer-active UA derivative, compound C-2028, forms GSH S-conjugate [8], yet it is still active against drug-resistant cancers [1], prompted us to expand our knowledge on this topic. It is well-documented that the mechanisms of GST-mediated conjugations are extremely variable and highly depend on the substrate and the GST class [9]. In addition, the activity of several human GSTs is strongly genetically determined due to gene deletions (null alleles) and single nucleotide polymorphisms (SNPs) [14]. The differential gene expression levels relate, for example, to the Alpha-, Mu-, and Pi-classes of GSTs (GSTA, GSTM, and GSTP, respectively), which are present at high levels in many human solid cancers (e.g., pancreatic, colorectal, breast, and bladder cancers) and have been indicated in many reports to be overexpressed in a wide range of sensitive and resistant cancer cells [15,16,17,18]. Accordingly, the affinity determination of a specific GST to a potential anticancer drug may reduce the phenomenon of drug resistance of neoplastic cells to the extent that it allows effective therapy.

The current study aimed to obtain enzyme kinetics for GSH conjugation of C-2028 in rat liver microsomes and cytosol. We also determined the optimal pH for the reaction. Then, we clarified the important GST isoenzymes involved in GSH conjugation of C-2028 produced in the human liver using selected human recombinant GSTs (hGSTs). The composition of the reaction mixtures was monitored chromatographically with UV-Vis detection. Using a colorimetric assay (MTT test), we initially assessed the cellular GST/GSH-dependent biotransformation of C-2028 in relation to cytotoxicity against Du-145 human prostate cancer cells in the presence or absence of the modulator of GSH biosynthesis (buthionine sulfoximine; BSO). Finally, nuclear magnetic resonance (NMR) spectroscopy was applied for structure determination of the earlier isolated and purified GSH-derived product of C-2028. We believe that the obtained results will translate into a better prediction of UA toxicity and improve anticancer therapy with these compounds that will minimize drug side effects.

## 2. Results

### 2.1. Formation of the GSH-Conjugated Metabolite of C-2028 in Rat Liver Microsomes and Cytosol as Well as with Recombinant Human GST Isoenzymes 

Rat liver microsomes (RLMs), rat liver cytosol (RCyt), or individual three recombinant human GST isoenzymes (A1-1, M1-1, and P1-1) purified from *Escherichia coli* expression systems were incubated for the indicated time with the tested C-2028 (0.05 mM) in the presence of GSH (5 mM) and subsequently analyzed by reversed-phase high-performance liquid chromatography (RP-HPLC). The typical elution profiles obtained from the chromatographic separation of incubation mixtures are shown in Figure 1. One main metabolite (t_R_ = 11.7 min) was detected in addition to C-2028 (t_R_ = 13.1 min), except for the reaction with hGSTA1-1 and hGSTP1-1. The intensity of its corresponding peak varied depending on the enzyme source used. It is interesting to note that this product was previously identified by liquid chromatography-tandem mass spectrometry (LC-MS/MS) analysis as monoglutathionyl-C-2028, and RLMs and RCyt catalyzed the formation of the same GSH S-conjugate of C-2028 (GS-C-2028) [8]. However, based on mass spectra alone, it was impossible to determine the proper site of attachment of the GSH molecule at that time. Good agreement in chromatographic retention time and UV-Vis spectrum confirmed the presence of this product also in reaction with hGSTM1-1, which preferentially catalyzed the GSH conjugation of C-2028 among all tested hGST isoforms. Available hGSTA1-1 and hGSTP1-1 did not show any conjugative reactions for C-2028, although our previous study [8] revealed minor involvement of Pi-class GST in C-2028 metabolism.

The peak eluted at t_R_ = 10.9 min (marked as X1) was reported in our previous studies as related to the product that contains a chromophore system of imidazoacridinone with the whole aminopropyl-N-methylaminopropylamino (-NH(CH_2_)_3_N(CH_3_)(CH_2_)_3_NH_2_) linker. In turn, the identity of the peaks eluted at t_R_ = 11.5 min (marked as X2), and t_R_ = 14.0 min (marked as X3) was unknown, but we observed that they appeared to a lesser extent only at higher concentrations of GSH (5 mM) without the participation of the enzyme. To provide additional data on these novels, likely non-enzymatic GSH-mediated products of C-2028, additional structure confirmation tools, such as high-resolution mass spectrometry or NMR, are needed.

We noticed that the formation of GS-C-2028 in RLMs was slightly slower than in RCyt. In the first case, almost 75% conversion of the substrate was achieved after ca. 30 min of reaction time, while the GSH-dependent enzymes of the cytosolic fraction needed only ca. 15 min to completely convert the parent compound into the product.

### 2.2. Effect of pH on GSH Conjugation of C-2028 in Rat Liver Microsomes and Cytosol

The rates of enzyme-catalyzed reactions often vary remarkably with pH. Generally, an enzyme is active only over a restricted pH range and usually has a marked optimum pH. For most enzymes, it is around 7 (neutral), although some enzymes work more efficiently at lower or higher pH [19]. GSTs, located mainly in hepatocytes, were structurally and functionally stable over a wide range of pH [20]. However, the extent of their selectivity enhancement is usually strongly related to the pH of the reaction medium [21]. Considering the above, RLMs or RCyt were incubated with C-2028 (0.05 mM) and GSH (5 mM) in the fixed pH of the reaction buffer.

**Figure 2:**
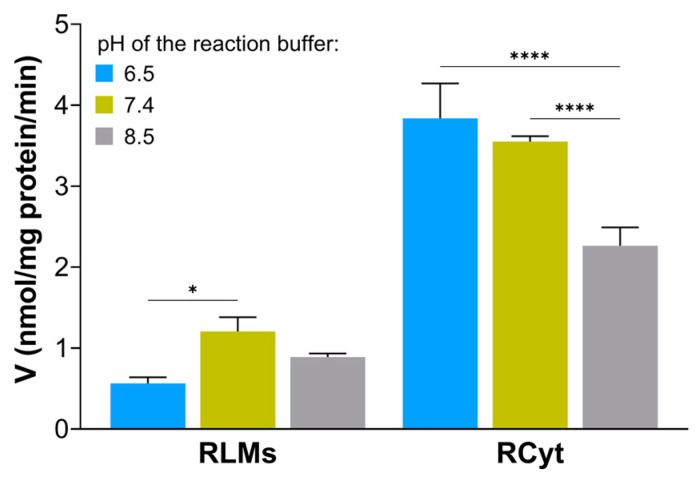
Influence of the pH of the 0.1 M Hepes buffer (reaction buffer) on the GST-dependent formation of GSH S-conjugate of C-2028 in rat liver microsomes (RLMs) and cytosol (RCyt). For experimental details, see the Materials and Methods section (Section 4). Data are expressed as means ± SD (standard deviation) of three independent determinations. Statistically significant differences were assessed using two-way ANOVA with Bonferroni multiple comparisons (* *p* < 0.05, **** *p* < 0.0001).

Enzymatic GSH conjugation reaction of C-2028 was obtained in all media. The surface areas under the chromatographic peaks of the GST/GSH-mediated product and substrate were measured in each case. Analysis of the pH-dependent rates for GS-C-2028 formation indicated that the highest increase in the product formation was observed at pH 7.4 for RLMs and pH 6.5 for RCyt (Figure 2). A clear decrease in C-2028 transformation, both in RLMs and RCyt, was observed in the reaction medium at pH 8.5. Considering that a lower pH value prevents UAs from self-aggregation and improves their solubility in aqueous media [5], we found pH 6.5 to be the best choice for achieving GST maximal activity for further analysis of enzyme kinetics.

### 2.3. Enzyme Kinetics of GSH Conjugation of C-2028 in Rat Liver Microsomes and Cytosol as Well as with Recombinant Human GSTM1-1 Isoenzyme

**Figure 3:**
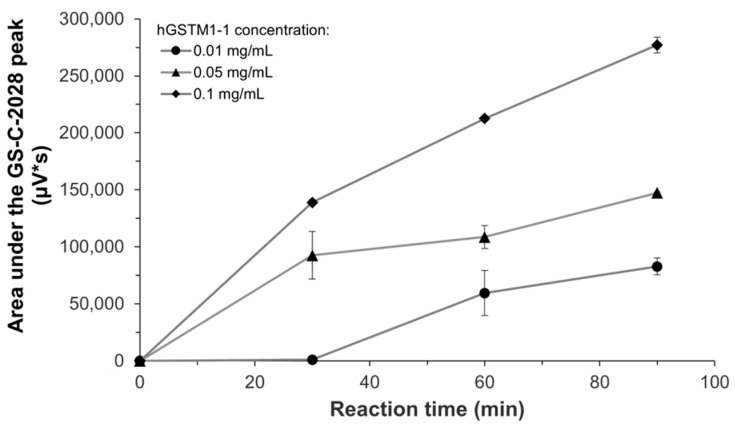
Area under the GSH S-conjugate of C-2028 peak against different concentrations of recombinant human GSTM1-1 as a function of reaction time. For experimental details, see the Materials and Methods section (Section 4). Data are expressed as means ± SD of two independent determinations.

Before proceeding to the comparative study of enzyme kinetics, we confirmed the linearity of the GS-C-2028 formation in RLMs and RCyt with respect to both the enzyme quantity (0.5, 1, 2 mg/mL) and reaction time (up to 30 min for RLMs and 15 min for RCyt). The influence of incubation time (30, 60, and 90 min) and the amount of recombinant hGSTM1-1 (0.01, 0.05, and 0.1 mg/mL) on the formation of GSH S-conjugate of C-2028 were also investigated. As shown in Figure 3, the formation of the product (measured as the area under the GS-C-2028 peak) was accelerated as the incubation time and enzyme concentration increased. At the lowest enzyme concentration used, the formation of the product required a reaction time of more than 30 min; after this, it increased slightly and gradually. After incubation for 90 min with 0.1 mg/mL GSTM1-1, the peak area of GS-C-2028 was significantly 3.4-fold higher compared with incubation with 0.01 mg/mL GSTM1-1.

**Figure 4:**
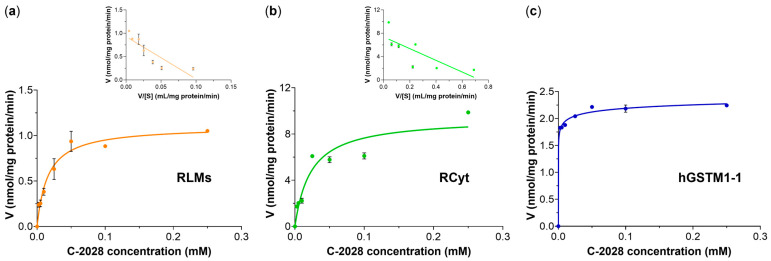
Kinetics of the GST-dependent formation of GSH S-conjugate of C-2028 in (**a**) rat liver microsomes (RLMs), (**b**) rat liver cytosol (RCyt), and (**c**) with recombinant human GSTM1-1 normalized to protein content as a function of C-2028 concentration. Eadie-Hofstee plots were shown as insets on the graphs. For experimental details, see the Materials and Methods section (Section 4). Data are expressed as means ± SD of three independent determinations.

Enzyme kinetic constants for the GST/GSH-mediated conjugation reaction of C-2028 were estimated using C-2028 concentrations 2.5–250 μM. The reactions were carried out under conditions where the concentration of the formed GS-C-2028 was linearly related to the reaction time (20, 5, or 60 min for RLMs, RCyt, or hGSTM1-1, respectively) and the amount of protein (1, 0.5, or 0.05 mg/mL for RLMs, RCyt, or hGSTM1-1, respectively). Plots illustrating reaction rate (*y*-axis) as a function of substrate concentration (*x*-axis) are shown in Figure 4. Data fitted to non-linear regression indicated that the formation of GSH S-conjugate of C-2028 in RLMs and RCyt followed Michaelis-Menten kinetics as we could observe a hyperbolic saturation profile of velocity of product formation (V) vs. substrate concentration ([S]) (Figure 4a,b). To define the kinetics in more detail, we also prepared the Eadie-Hofstee plots (V vs. V/[S]) [22]. A not very clear linear fit (R^2^ = 0.73 and 0.59 for RLMs and RCyt, respectively) may mean biphasic kinetics [23] that arises from the involvement of multiple GSTs, having different kinetic properties, in C-2028 metabolism. The obtained enzyme kinetic parameters, estimated by fitting the derived data to the Michaelis-Menten model, were reported in Table 1. The maximal velocity of GS-C-2028 formation (V_max_) was ca. 8.5-fold higher for RCyt than RLMs. The Michaelis constants (K_M_) also differed clearly between these two investigated GST sources. A lower K_M_ value estimated for RLMs pointed out the better affinity between the substrate and the present GSTs.

Interestingly, the kinetic of the hGSTM1-1-catalyzed reaction proceeded according to a slightly different kinetic pattern that we could describe as a sigmoid-like model. The plot shown in Figure 4c implies that at the lowest substrate concentrations, the reaction rate increased sharply and practically did not change. The enzyme quickly bound to the substrate and held it in a way that allowed the reaction to occur at almost maximal reaction velocity. Consequently, sigmoidal substrate saturation curves in the Michaelis-Menten mechanism were observed. No substrate inhibition profile was found in any studied case.

### 2.4. Assessment of Cytotoxicity of the GSH S-Conjugate of C-2028 toward Du-145 Human Prostate Cancer Cells

This study stage aimed to determine whether modulation of the cellular reduced GSH level influences the cellular sensitivity to C-2028. Du-145 human prostate cancer cell line was treated with BSO, a specific inhibitor of γ-glutamylcysteine synthetase [24]. Afterwards, the C-2028-mediated cytotoxicity was established using the MTT cytotoxicity assay.

The data presented in Table 2 correspond to the concentration of the C-2028 compound required to inhibit the growth of untreated and BSO-pretreated cells by 50% (IC_50_). C-2028 exhibited high cytotoxic activity against Du-145 cells. In contrast, a marked decrease in the cytotoxicity of C-2028 against the Du-145 cell line was observed after GSH depletion by exposure of cancer cells to BSO. This was associated with the reduction of the intracellular amount of monoglutathionyl-C-2028, thus suggesting a possible impact of this product on the overall cytotoxic activity of C-2028.

### 2.5. Characterization of the GSH S-Conjugate of C-2028 by NMR Spectroscopy

The initial structural identification of GS-C-2028 was acquired by LC-MS/MS [8]. The positive ion mass spectrum of this product showed a stable molecular ion at *m*/*z* = 846. However, without an authentic standard and based on mass spectra alone, it was not possible to determine the site of attachment of the GSH molecule to the C-2028 molecule.

The full structure elucidation of the GSH S-conjugate of C-2028 by NMR required the isolation of a relatively large amount of a pure product. Therefore, it was biosynthesized and purified from scale-up incubation of C-2028 with RCyt supplemented with an excess of GSH.

**Figure 5:**
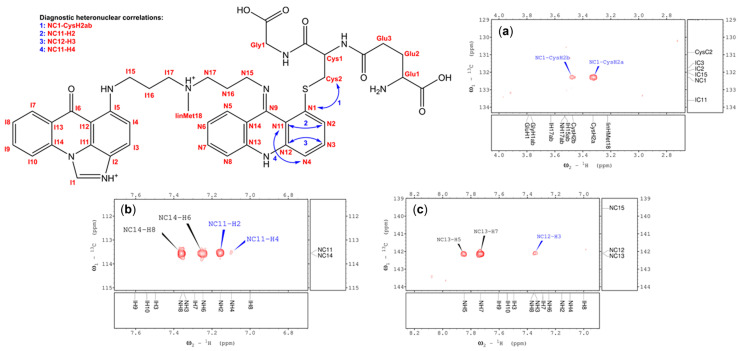
NMR-derived structure of the resulting GSH S-conjugate of C-2028. Panels (**a**–**c**) show fragments of the ^1^H-^13^C HMBC spectrum (recorded at 55 °C), which contain diagnostic proton-carbon correlations enabling unambiguous localization of the peptide moiety, according to the bidirectional arrows displayed at the GSH S-conjugate’s structure.

Thorough analysis of the ^1^H-^1^H DQF-COSY, ^1^H-^1^H NOESY, ^1^H-^13^C HSQC, and ^1^H-^13^C HMBC experiments, recorded for the obtained GSH-derived product of C-2028 at 35 °C, has evidenced that during the discussed conjugation reaction a GSH moiety indeed attaches to the nitroacridine ring system of C-2028 via sulfide bond. Careful examination of the protonic spin systems observed in the NMR spectra narrowed the possible attachment positions of GSH moiety to merely two options, i.e., N1 or N4 (Figure 5). Unfortunately, since the proton resonances of the N1-N4 fragment were substantially broadened, presumably caused by a bulky GSH substituent, no diagnostic ^1^H-^13^C correlations were found in the HMBC spectrum. Nevertheless, at 55 °C, the examined GSH S-conjugate was surprisingly stable. Hence, a second set of HSQC and HMBC spectra was registered. At that temperature, the N1-N4 resonances were notably sharpened and, therefore, four vital ^1^H-^13^C correlations have been observed: CysH2ab-NC1, NH2-NC11, NH3-NC12 and NH4-NC11 (Figure 5). These heteronuclear couplings have revealed the exact position of the GSH moiety within the resulting metabolite, i.e., N1. 

In the end, it was unambiguously proven that GSTs present in RCyt enable a GSH molecule to substitute the nitro group attached to the nitroacridine ring system of C-2028. This result is coherent with our previous speculations on the structure of the GST/GSH-related metabolite of C-2028 [8]. The complete ^1^H and ^13^C NMR spectral assignment of the obtained conjugate was presented in Table S1 in Supplementary Materials. Supplementing fragments of the recorded NMR spectra were also included in Figures S1 and S2.

## 3. Discussion

GST-catalyzed GSH conjugation is a major drug elimination reaction in humans [9]. Many classes of anticancer drugs are substrates for this pathway. There are many aspects to consider in this topic. GST/GSH-dependent biotransformation of activated drugs usually results in the loss of their biological activity (via detoxification pathway). However, in some cases, GSH may also generate more activity than parent drugs or toxic compounds (via the bioactivation pathway) [10]. For these reasons, the study on GSH conjugation of a drug candidate is pivotal for determining its therapeutic efficacy or toxicological activity. 

Our preliminary studies reported that the metabolic pathway of anticancer C-2028 involves the action of GST to conjugate GSH with a compound [8]. We found that in excess of reduced GSH, C-2028 was extensively metabolized in RLMs and RCyt to one GSH S-conjugate (Figure 1), whose structure was tentatively identified by LC-MS/MS analysis. Although we did not observe the formation of the GSH-derived product of C-2028 in human liver subfractions, this discrepancy may have been due to species-specific differences and a lack of the specific GST isoforms responsible for the formation of GS-C-2028 in human liver microsomes and cytosol.

GST conjugative enzymes are present in different subcellular compartments, including cytosol, mitochondria, endoplasmic reticulum, nucleus, and plasma membrane [25]. The current study provided a detailed investigation of the in vitro GST/GSH conjugation of C-2028 in RLMs and RCyt, as well as by the selected recombinant human GST isoenzymes. Consistent with previous results, we obtained a greater degree of C-2028 conversion to GS-C-2028 with the GSTs of the cytosolic than the microsomal fraction. The observed variability in GST/GSH-mediated conjugation of C-2028 resulted most from great individual differences in the amount and/or catalytic activity of particular GSTs in the used RLMs and RCyt. Due to this observation, the liver cytosolic fraction was further used as the biocatalyst for the synthesis of GS-C-2028 for NMR measurements. Continuing, we conducted experiments in reaction buffers that differed in pH. Literature reports that GSTs catalyze the conjugation of GSH with a xenobiotic by lowering the pK_a_ of GSH from ca. 9 to 6.4–6.7 and stabilizing the thiolate anion (GS^−^) through hydrogen bonding [26]. The pH-dependent efficiency of GST/GSH-mediated conjugation reaction in human liver S9 fraction was observed, i.a., by Li et al., who were studying the transformation of AZD1979 (a melanin-concentrating hormone receptor 1 antagonist) to a series of GSH-related metabolites [27]. In our study, the formation of GS-C-2028 in RLMs and RCyt had similar yields at pH 6.5 and 7.4 and was significantly higher than at pH 8.5. The optimal pH of 6.5–7.4 for the formation of the GSH S-conjugate of C-2028 satisfied the formation of GS^−^ via the GST-bound GSH (pK_a_ 6.4–6.7). Finally, for further experiments, we stayed with Hepes buffer at pH 6.5 as we determined a high stimulation of the catalytic activity of the GSTs, especially in RCyt. It should also be mentioned that a lower pH value better prevents UA derivatives from self-aggregation in aqueous media [5]. In both rat liver subfractions, we found that GS-C-2028 formation followed Michaelis-Menten kinetics (Figure 4a,b). The values of enzyme kinetic parameters indeed confirmed that liver cytosolic GSTs were more efficient than microsomal at catalyzing the C-2028-GSH conjugation reaction. Since the data points in the Eadie-Hofstee plots were not homogeneously distributed, we proposed biphasic kinetic profiles. This is commonly observed in liver preparations where multiple cytochrome P450 isoforms catalyze diverse reactions [28]. Accordingly, our results may mean that multiple GST isoforms, characterized by markedly differing substrate binding affinities, may be responsible for the formation of the observed product. Therefore, a very important concern was to determine which individual GST isoforms are involved in C-2028 metabolism.

In the present work, GSH conjugation of C-2028 was evaluated using three selected recombinant human GST isoforms that belong to the most abundant soluble cytosolic GSTs and characterize overlapping substrate specificity [29]. GSTA, GSTM, and GSTP attract interest mainly because they are highly expressed in different cancer tissues, such as pancreatic, colorectal, breast, and bladder cancers [15,16,17,18]. Moreover, their altered expression has been implicated in hepatic, cardiac, and neurological diseases [25]. Our initial screening indicated that among three tested hGST isoforms, only hGSTM1-1 catalyzed GS-C-2028 formation to the extent that it was detectable (Figure 1). This finding signifies that the presence of GSTM1-1 in tumors may contribute to enhanced detoxification of UA and hence the development of drug resistance. Looking at kinetics for hGSTM1-1-mediated GSH conjugation of C-2028 (Figure 4c), we observed almost instantaneous substrate consumption by the enzyme that caused a rapid increase in the velocity of the reaction until V_max_ was achieved. The data points fit best with a sigmoidal regression rather than the Michaelis-Menten equation. It can happen when the enzyme has cooperative subunits, and additional active sites could speed up the reaction [30]. Sigmoidal dependence of the initial rates on substrate concentration can indicate positive cooperativity, which refers to the observation that binding one substrate molecule to the enzyme at one binding site promotes the binding of an additional molecule at other sites.

GSTs are dimeric enzymes usually consisting of two identical subunits of separate domains [31]. The GSH-binding site (G-site) of mammalian GSTs is structurally similar, whereas the shape of the hydrophobic substrate-binding pocket (H-site) differs substantially among various GSTs and, therefore, greatly increases their diversity. Considering the crystallographic structure of the mammalian Mu-class GSTs, it is a homodimeric protein in which each GST subunit contains an independent catalytic site [29]. A unique feature of Mu-type GSTs is the presence of a large Mu-loop between β-2-sheet and α-2-helix. This creates a deeper cleft in the active site of the enzyme than the one found in, e.g., the Pi-class GSTs. Thus, the space to recognize and bind voluminous substrates is extended [32]. So, this structural feature may be responsible for the enzyme’s ability to bind C-2028 molecules in the active sites.

With the application of the NMR technique, we determined the full structure of the GSH S-conjugate of C-2028. It was revealed that during the GST-mediated conjugation, a GSH molecule substitutes the nitro group of the nitroacridine ring system of C-2028 exactly at the N1 position (Figure 5). Hence, synthesizing the compound standard necessary for further advanced biological and bioanalytical investigations will be achievable. Finally, based on the known structures of C-2028, its GSH S-conjugate, and GST active centers, we can propose a likely manner of C-2028 binding to the enzyme. It can be supposed that one of the C-2028 rings binds non-covalently (possibly through hydrogen bonding or Van der Waals interactions) in one active center. The one containing a nitro group is donated to the second active center of the same enzyme molecule but in such a way that the carbon atom, the site of the GSH attack, is exposed outside. Further, the length of the linker connecting the two rings of the C-2028 molecule is probably similar to the distance between the centers of both active centers of the enzyme.

Next, it was not known whether GS-C-2028, like the parent compound (C-2028), also exhibits any substantial biological properties. Here, we demonstrated the first study that provided insight into cellular GST/GSH-dependent biotransformation of C-2028 in relation to cytotoxicity against cancer cells. In a preliminary experiment, Du-145 human prostate cancer cells were pretreated with a modulator of GSH biosynthesis (BSO), which resulted in the reduction in the intracellular amount of GSH S-conjugate of C-2028. If its formation is a real detoxification pathway, we should observe an increase in the cytotoxicity of the compound in BSO-pretreated cells. However, our study revealed that cellular GSH depletion decreased the sensitivity of Du-145 cancer cells to C-2028 (Table 2). In line with this result, we can speculate on the GSH conjugation of UA as a bioactivation reaction, as the GSH-derived product of C-2028 may also be cytotoxic for the cells. Thus, it appears very interesting to evaluate C-2028-GSH S-conjugate biological activity in detail. Future studies should include studies on the influence of other modulators of GST/GSH-related processes (i.e., inhibitors of GST activity or GSH-conjugate efflux pumps) toward GSH-dependent biotransformation of UA and GSH S-conjugate transport. Nevertheless, it should be noted that GSH loss is an active phenomenon regulating the redox signaling events modulating cell death activation and progression [33]. Therefore, the combination of this factor and other unknown factors may be critical for the onset of C-2028 cytotoxicity. 

Besides the GST-catalyzed reaction, C-2028 was also found to undergo conjugation with GSH in a non-enzymatic way. This indicates that in cells with high levels of GSH and low drug concentrations, non-enzymatic reaction may be relatively important compared to enzymatic conjugation rates. Hence, the actual contribution of GSTs to C-2028 biotransformation in cancer cells under physiological conditions needs to be addressed in further research.

## 4. Materials and Methods

### 4.1. Chemicals

Unsymmetrical bisacridine 9-{N-[(imidazo[4,5,1-de]-acridin-6-on-5-yl)aminopropyl]-N-methylaminopropylamino}-1′-nitroacridine (C-2028) was synthesized and purified in our laboratory according to the method described in [1]. D, L-Buthionine sulfoximine (BSO; ≥99%), 1-chloro-2,4-dinitrobenzene (CDNB; ≥99%), 3-(4,5-dimethyl-2-thiazolyl)-2,5-diphenyl-2H-tetrazolium bromide (MTT; 98%), dimethyl sulfoxide (DMSO), formic acid (HCOOH; ≥98%), L-glutathione reduced (GSH; minimum 99%), hydrochloric acid (HCl), magnesium chloride (MgCl_2_; minimum 98%), and 2-[4-(2-hydroxyethyl)piperazin-1-yl]ethane-1-sulfonic acid (Hepes; ≥99.5%) were purchased from Sigma-Aldrich (St. Louis, MO, USA). Methanol (CH_3_OH; gradient grade for liquid chromatography) was obtained from Merck KGaA (Darmstadt, Germany). Potassium hydroxide (KOH) was delivered from Chempur (Piekary Śląskie, Poland). All other chemicals and solvents were commercially available, of analytical grade, and used without further purification. Ultrapure water (H_2_O; resistivity 18.2 MΩ·cm at 25 °C), used in all the experiments, was passed through a Milli-Q IQ 7005 Water Purification System from Merck KGaA. 0.1 M Hepes buffer solution was prepared in ultrapure H_2_O, and pH was adjusted to 6.5, 7.4, or 8.5 values with 1 M KOH. The stock solutions of C-2028 and GSH were prepared in ultrapure H_2_O at concentrations of 10 mM and 100 mM, respectively. The 100 mM stock solution of CDNB was made in DMSO, and on the day of the experiment, it was dissolved in ultrapure H_2_O to reach the concentration of 10 mM.

### 4.2. Enzymes

Pooled IGS Sprague-Dawley rat liver microsomes (RLMs; protein concentration 20 mg/mL) and cytosol (RCyt; protein concentration 10 mg/mL) were purchased from Sekisui Xenotech, LCC (Kansas City, KS, USA; through Tebu-Bio, Le Perray-En-Yvelines, France). Recombinant human glutathione S-transferase isoforms A1-1 (hGSTA1-1), M1-1 (hGSTM1-1), and P1-1 (hGSTP1-1), produced and purified from *Escherichia coli*, were obtained from Sigma-Aldrich. The stock solution concentrations of hGSTs were as described in the data sheets provided by the manufacturer. All enzymes were stored at −80 °C before usage. CDNB was used as a GST standard substrate [34] to determine the catalytic activity of RLMs, RCyt, and hGSTs (positive control). The reactions were carried out by incubating 0.1 mM CDNB with 0.2 mM GSH and the appropriate number of enzymes (1 mg/mL for RLMs and RCyt, 0.05 mg/mL for hGSTs) in 0.1 M Hepes buffer (pH 6.5) for 10 min in a water bath at 37 °C. The reaction was terminated by ice-cold 1 M HCl (10:90, *v*/*v*). The reaction solution was centrifugated (13,400 rpm for 5 min), and the supernatant was directly analyzed by RP-HPLC with UV-Vis detection. The activity of GSTs with CDNB was determined spectrophotometrically by monitoring the formation of a dinitrophenyl thioether (DNB-SG) detected at 340 nm. All of the measurements were corrected for the spontaneous non-enzymatic rate of reaction between GSH and CDNB. Ultimately, it had a negligible percentage in the process of GSH conjugation to the CDNB molecule.

### 4.3. Effect of pH on the Formation of the GSH-Conjugated Metabolite of C-2028 in Rat Liver Microsomes and Cytosol

To optimize the reaction conditions, we decided to examine the effect of environmental pH on the catalytic activity of GSTs involved in the GSH conjugation of C-2028. For this aim, we carried out investigations by testing three pH values for the Hepes buffer: 6.5, 7.4, and 8.5.

C-2028 (0.05 mM), MgCl_2_ (2 mM), and RLMs or RCyt (1 mg/mL) were added to 0.1 M Hepes buffer (pH 6.5, 7.4, or 8.5), and the mixture was pre-incubated for 3 min in a water bath at 37 °C. Afterwards, GSH (5 mM) was added in a total volume of 50 µL, and the samples were further incubated for 10, 20, and 30 min. Control experiments in the absence of GSH were run in parallel. Additionally, stability control of the investigated compound was incubated without enzymes to differentiate between enzymatic and non-enzymatic reactions. After the reactions had been terminated by the addition of ice-cold 1 M HCl (1:9, *v*/*v*), samples were kept in ice for 10 min and centrifuged (13,400 rpm for 5 min); a total of 40 µL of the supernatant was injected into the RP-HPLC column.

Enzyme kinetics for GST-catalyzed GSH conjugation were evaluated by incubation of C-2028 (2.5–250 μM) with RLMs (1 mg/mL) or RCyt (0.5 mg/mL) in the reaction buffer at pH 6.5 for 20 and 10 min, respectively. Samples for HPLC analyses were prepared identically, as mentioned above. Assays were performed in triplicate.

As a standard of GSH S-conjugate of C-2028 was not available, the determination of the product concentration was based on the assumption that it had a molar extinction coefficient similar to that of the studied UA. Thus, we could estimate the relative GS-C-2028 amount according to calibration curves derived from the peak areas of C-2028 samples over the 2.5–250 μM drug concentrations. The assay was done in triplicate. The number of product moles (n) was calculated from the following equation (Equation (1)):

n = [(P_0_ − P_t_) − b]/a, (1)

where: P_0_ is an area under the C-2028 peak at the start of the reaction (t = 0), P_t_ is an area under the C-2028 peak after t min of incubation, b the *y*-intercept of a graph, and a refers to a calibration curve slope.

The analysis of the composition of the obtained reaction mixtures was carried out by RP-HPLC method using Waters Associates HPLC system (Waters Co., Milford, MA, USA) equipped with a model 600E system controller, a model 7725i Rheodyne injector, 717 plus Autosampler, and a model 2996 photodiode array detector (DAD) controlled with Empower 3 version software (Waters Co., Milford, MA, USA). Chromatographic separation of C-2028 or CDNB and their GSH S-conjugates was achieved on a Suplex pKb-100 column (5-µm particle size, 150×4.6 mm i.d.; Supelco, Inc., Bellefonte, PA, USA), at a flow rate 0.6 mL/min. The mobile phase consisted of a mixture of (A) ultrapure H_2_O with 0.1% HCOOH (*v*/*v*) and (B) CH_3_OH with 5% ultrapure H_2_O (*v*/*v*). The following linear gradient elution was applied: 15% B from 0–15 min, 100% B from 15–17 min, 100% B from 17–17.5 min, and 15% B from 17.5–5 min (returning to initial conditions). The autosampler and the HPLC column were kept at 4 °C and room temperature, respectively. The eluates were monitored with UV-Vis detection at 420 nm (for C-2028 samples) or 325 nm (for CDNB samples), respectively.

### 4.4. In Vitro Incubation of C-2028 with Recombinant Human GST Isoenzymes

To assay C-2028 GST-mediated GSH conjugation, C-2028 was also incubated with the selected recombinant human GSTs and supplemented with GSH. The reaction mixture contained C-2028 (0.05 mM), MgCl_2_ (2 mM), GSH (5 mM), and a single hGST (0.01, 0.05, or 0.1 mg/mL) in 0.1 M Hepes buffer (pH 6.5) in a total volume of 30 μL. Samples were incubated for 60 min in a water bath at 37 °C. Control experiments in the absence of GSH were run in parallel. After incubation, samples were processed as described above by protein precipitation and centrifugation. The formation of GSH S-conjugate of C-2028 was monitored by RP-HPLC.

hGSTM1-1-mediated conjugation of C-2028 (0.05 mM) was further investigated at different GST concentrations (0.01, 0.05, and 0.1 mg/mL) in the presence of GSH (5 mM) during incubation for 30, 60, and 90 min. Assays were performed in duplicate. Enzyme kinetic for GSH conjugation by hGSTM1-1 was evaluated by incubation of the recombinant enzyme (0.05 mg/mL) with C-2028 (2.5–250 μM) in the presence of GSH (5 mM) for 60 min. Assays were performed in triplicate. Samples for HPLC analyses were prepared identically, as mentioned above.

### 4.5. Cell Culture

Du-145 human prostate cancer cell line was purchased from the American Type Culture Collection (ATCC; Manassas, VA, USA). Cells were grown in an Eagle’s Minimum Essential Medium (EMEM; Sigma-Aldrich, USA) supplemented with 10% fetal bovine serum (FBS; Biowest, Riverside, MO, USA), 100 µg/mL of streptomycin (Sigma-Aldrich, China), and 100 units/mL of penicillin (Sigma-Aldrich, Israel). Cells were incubated in a humidified atmosphere containing 5% CO_2_ at 37 °C. Experiments were performed with cells in the exponential phase of growth.

### 4.6. MTT Cytotoxicity Assay

The sensitivity of Du-145 human prostate cancer cells to exposure to C-2028 in the presence or absence of the modulator of GSH biosynthesis (BSO) was measured using a 3-(4,5-dimethyl-2-thiazolyl)-2,5-diphenyl-2H-tetrazolium bromide (MTT) cytotoxicity assay. Briefly, cells were plated in triplicate in 24-well plates at a seeding density of 3 × 105 cells/well in 2 mL of supplemented cell culture medium. To examine the effect of GSH depletion by BSO on GSH-dependent biotransformation C-2028 and cytotoxicity of C-2028, cells were cultured in the presence of 50 μM BSO for 24 h.

After 24 h of preincubation, cells were washed in 1× phosphate-buffered saline (PBS; pH 7.4). Next, the fresh medium containing 50 μM BSO was added. Controls were incubated with medium mixed with sterile ultrapure H_2_O. After 30 min preincubation with the modulator, cells were treated with different concentrations of C-2028 (up to 2.5 μM). The 10 mM stock solution of the compound and its dilutions were prepared in sterile ultrapure H_2_O. Following 24 h of incubation, 200 µL of MTT solution (4 mg/mL in ultrapure H_2_O) was added to each well, and cells were allowed to stain for 4 h in the cell culture incubator (5% CO_2_, 37 °C). Then, media containing MTT solution were removed, and formazan crystals were dissolved in 0.5 mL of DMSO, shaking the plates for 30 min. Finally, the absorbances of solutions at 540 nm were measured using a microplate reader (iMarkTM Microplate Absorbance Reader; Bio-Rad, Hercules, CA, USA). Cytotoxicity was calculated via GraphPad Prism 9 program (trial version 9.5.1.733; GraphPad Software Inc., San Diego, CA, USA) using a point-to-point function and was expressed as IC_50_ value estimated from full dose-response curves (drug concentrations inducing a 50% reduction of cell number in comparison with untreated control cells cultured in parallel). Results were obtained using three independent experiments.

### 4.7. Biosynthesis and Purification of the GSH-Conjugated Metabolite of C-2028

RCyt was used here as the biocatalyst for the synthesis of GSH S-conjugate of C-2028. The reaction mixture contained C-2028 (0.3 mM), RCyt (1 mg/mL), MgCl_2_ (2 mM), and GSH (5 mM) in 0.1 M Hepes buffer (pH 6.5) in a total volume of 15 mL. The sample was incubated for 40 min in a water bath at 37 °C in a 50 mL falcon tube vortexed periodically. The progress of the reaction was monitored by RP-HPLC with UV-Vis detection. After incubation, the reaction mixture was quenched using ice-cold CH_3_OH (1:2, *v*/*v*; 45 mL), and the solution was allowed to stand in a refrigerator at 4 °C for 60 min. Following two centrifugations, each for 30 min at 9500 rpm and 4 °C, the combined supernatants were transferred to a round-bottomed flask and concentrated on a rotary evaporator (Hei-Vap Core; Heidolph Instruments GmbH & Co. KG, Schwabach, Germany) at 37 °C. After the removal of the organic solvents, the aqueous fraction was subjected to fractionation by RP-HPLC under conditions identical to those mentioned above. The HPLC system consisted of an LC-20AD prominence liquid chromatograph, a CBM-20A system controller with a SIL-10AF autosampler, an FRC-10A fraction collector, and SPD-M20A prominence diode array detector controlled with LabSolutions software (Schimadzu Corp., Kyoto, Japan). The GS-C-2028-containing fractions were combined, concentrated using a centrifuge concentrator (Eppendorf Poland Sp. z o.o., Warsaw, Poland), and dried in an argon stream. Finally, the sample was dissolved in 0.6 mL of H_2_O-deuterium oxide (D_2_O) mixture (9:1, *v*/*v*). The final solution was transferred to a 5-mm NMR tube for NMR analyses.

### 4.8. NMR Characterization of the GSH-Conjugated Metabolite of C-2028

NMR spectra were acquired for isolated GSH S-conjugate of C-2028 on a 700 MHz Bruker Avance III HD spectrometer equipped with a QCI CryoProbe (Bruker Corp.; Billerica, MA, USA). All the experiments were performed in H_2_O-D_2_O (9:1, *v*/*v*) solvent system in 10 mM cacodylate buffer (pH 5.0) at 35 °C and/or 55 °C with a sample concentration of ca. 0.62 mg/mL. The δH/δC chemical shifts were reported in (ppm) units using ^1^H residual resonance of water (4.687 ppm) as the internal standard.

The 1D-^1^H NMR spectra were collected with a digital resolution of 0.5 Hz. The ^1^H 90° pulse length was 7.6 µs. The 2D-^1^H-^1^H NMR spectra were measured in the phase-sensitive mode with a spectral width of 6313 Hz. A DQF-COSY spectrum was acquired at 35 °C in a 2048 × 256 matrix with eight accumulations per increment and was processed in a 2K × 1K matrix. A NOESY spectrum was acquired at 35 °C with a mix time of 500 ms in a 2048 × 256 matrix with 112 accumulations per increment and was processed in a 2K × 1K matrix.

The 2D-^1^H-^13^C-HSQC and -HMBC experiments were performed with pulse field gradients. Edited HSQC spectra were acquired at 35 °C and 55 °C in the phase-sensitive mode with ^1^J_(CH)_ set to 145 Hz. The spectral windows for the ^1^H and ^13^C axes were 6313 Hz and 29,177 Hz, respectively. Data were collected in a 2048 × 256 matrix with 64 accumulations per increment and processed in a 2K × 1K matrix. The HMBC spectra were acquired at 35 °C and 55 °C in absolute value mode with ^n^J_(CH)_ set to 12 Hz or 20 Hz. The spectral windows for the ^1^H and ^13^C axes were 7716 Hz and 36,992 Hz, respectively, or 6313 Hz and 7958 Hz, respectively. The data were collected in a 2048 × 256 matrix with 200 accumulations per increment and processed in a 2K × 1K matrix.

### 4.9. Data Analysis

All experiments were carried out with at least three independent replicates, and the results were expressed as means ± standard deviation (SD). C-2028-GSH S-conjugate formation rates were mathematically adjusted to the individual conjugation activity of microsomal, cytosolic, and recombinant GST enzymes, as given by the manufacturer.

The statistical significance of pH’s effect on the GSH S-conjugate formation in RLMs and RCyt was determined by applying the two-way ANOVA analysis of variance for multiple comparisons. The Bonferroni test was used to determine the source of significance where appropriate. Statistical significance was set at * *p* < 0.05, **** *p* < 0.0001.

For the determination of the enzyme kinetic constants (K_M_ and V_max_), the data were fitted to the Michaelis-Menten (hyperbolic) model and further analyzed using Eadie-Hofstee (linear) plots. The coefficient of determination (R^2^) and visual inspection of the Eadie-Hofstee plots were used to verify the quality of a fit to a model of enzyme kinetics. Enzyme kinetic parameters were estimated using the GraphPad Prism 9 program (trial version 9.5.1.733; GraphPad Software Inc., San Diego, CA, USA) for Michaelis-Menten (Equation (2)):

V = V_max_ × [S]/(K_M_ + [S]), (2)

where: V is the initial velocity, V_max_ is the maximal velocity, K_M_ is the Michaelis constant, and [S] is the substrate concentration.

## 5. Conclusions

GST/GSH system participates in the metabolism of natural substrates and drug metabolism in the patient organism. Therefore, this has a significant impact on the final effectiveness of the applied therapy procedures. Our current results confirmed the importance of GST/GSH conjugation in the biotransformation of C-2028, which must be considered in humans when planning a treatment strategy. We demonstrated that C-2028 was preferentially metabolized by liver-specific GSTM1-1 isoenzyme, which may also apply to human hepatocytes. From our perspective, more information is still needed on the cellular GSH-dependent biotransformation of C-2028 and the transport of the main monoglutathionyl metabolite in relation to cytotoxicity in various cancer cell lines. Measurements of both parent and GSH-related species will be necessary to correlate pharmacokinetics with pharmacological activity. It remains unclear and represents an important theme for further studies.

## Figures and Tables

**Table 1:** Enzyme kinetic parameters of GS-C-2028 formation in rat liver microsomes (RLMs) and cytosol (RCyt). Data are expressed as means ± SD of three independent determinations.

Source of GSTs	K_M_ (µM)	V_max_ (nmol/mg Protein/min)
RLMs	15.93 ± 4.79	1.10 ± 0.09
RCyt	22.88 ± 12.42	9.40 ± 1.42

**Table 2:** The effect of buthionine sulfoximine (BSO) modulator of GSH-related processes on the cytotoxicity of C-2028 against Du-145 human prostate cancer cells as measured by MTT-formazan dye formation. IC_50_ values are expressed as means ± SD of three independent determinations. Statistical significance difference (Student’s *t*-test) between the cytotoxicity of C-2028 in the cells untreated and pretreated with BSO was set at * *p* < 0.05.

Treatment	IC_50_ (µM)
No modulator	0.011 ± 0.008
BSO	0.061 ± 0.005 *

## Data Availability

All data generated or analyzed during this study are included in this published article and its Supplementary Material files. The raw data is available on request from the corresponding author.

## Supplementary Materials

The following supporting information can be downloaded at https://www.mdpi.com/article/10.3390/molecules28196812/s1, Figure S1: Edited ^1^H-^13^C HSQC spectrum of the GSH S-conjugate of C-2028; Figure S2: Fragment of the ^1^H-^1^H NOESY spectrum of the GSH S-conjugate of C-2028, displaying dipolar couplings between the protons of C-2028 and the GSH moiety, marked in blue; Table S1: ^1^H and ^13^C NMR spectral assignment of the GSH S-conjugate of C-2028, based on DQF-COSY, NOESY, HSQC, and HMBC spectra recorded at 55 °C. All the positions are listed as presented in Figure 5. The chemical shifts are reported in (ppm) units using ^1^H residual resonance of water (4.687 ppm) as the internal standard.

## Sample Availability

A sample of the compound C-2028 is not available from the authors.
