# Supplementary material for: In Vitro Enzyme Kinetics and NMR-Based Product Elucidation for Glutathione S-Conjugation of the Anticancer Unsymmetrical Bisacridine C-2028 in Liver Microsomes and Cytosol: Major Role of Glutathione S-Transferase M1-1 Isoenzyme
Source: Molecules. 2023 Sep 26;28(19):6812. doi: 10.3390/molecules28196812 (PMC10574777; doi:10.3390/molecules28196812)
Supplement: Supplementary file 1 [file molecules-28-06812-s001.zip › molecules-2613441-supplementary.docx]

Supplementary Materials

for

In Vitro Enzyme Kinetics and NMR-Based Product Elucidation for Glutathione S-Conjugation of the Anticancer Unsymmetrical Bisacridine C-2028 in Liver Microsomes and Cytosol: Major Role of Glutathione S-Transferase M1-1 Isoenzyme

**
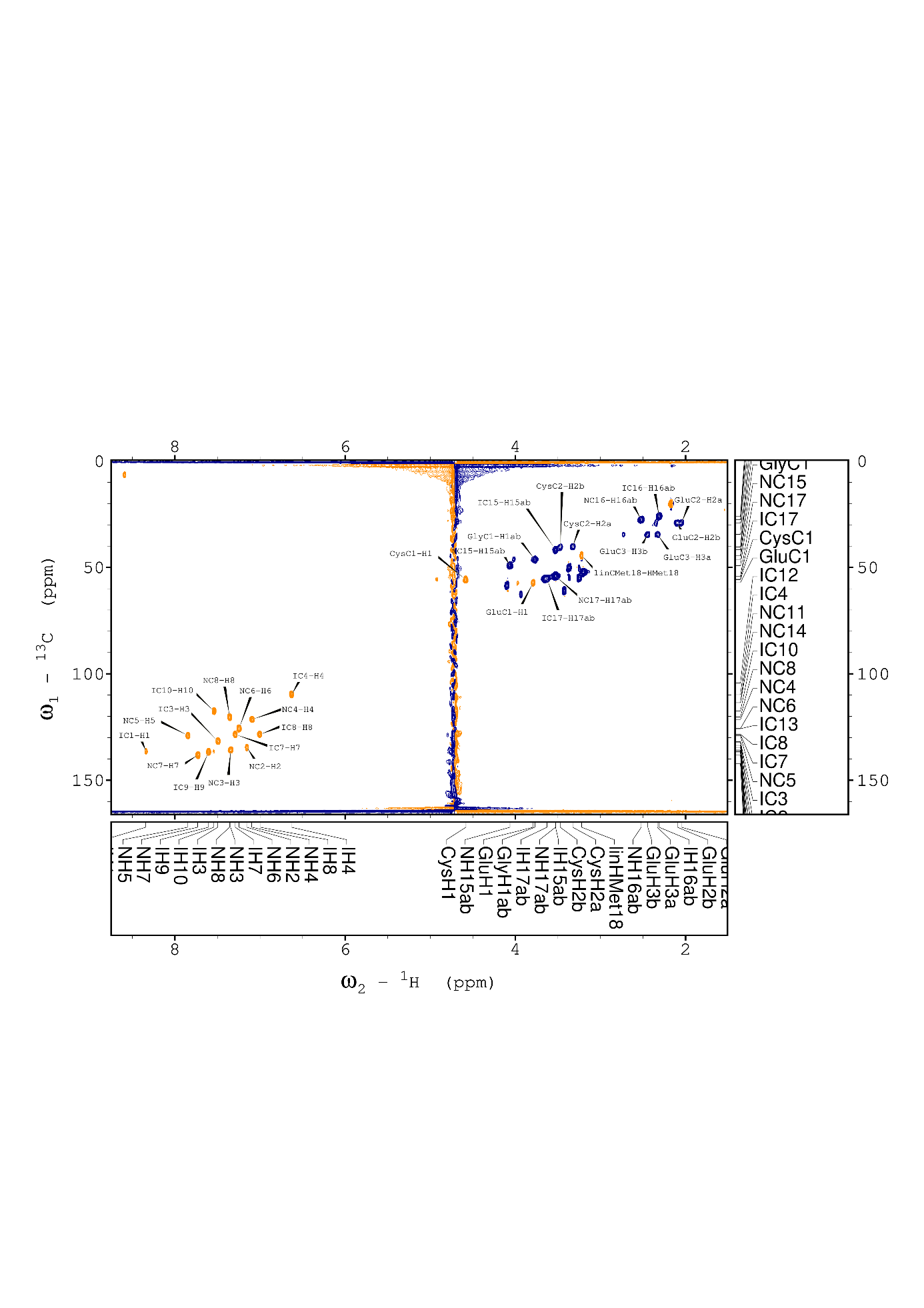
Figure S1.** Edited ^1^H-^13^C HSQC spectrum of the GSH S-conjugate of C-2028.

**
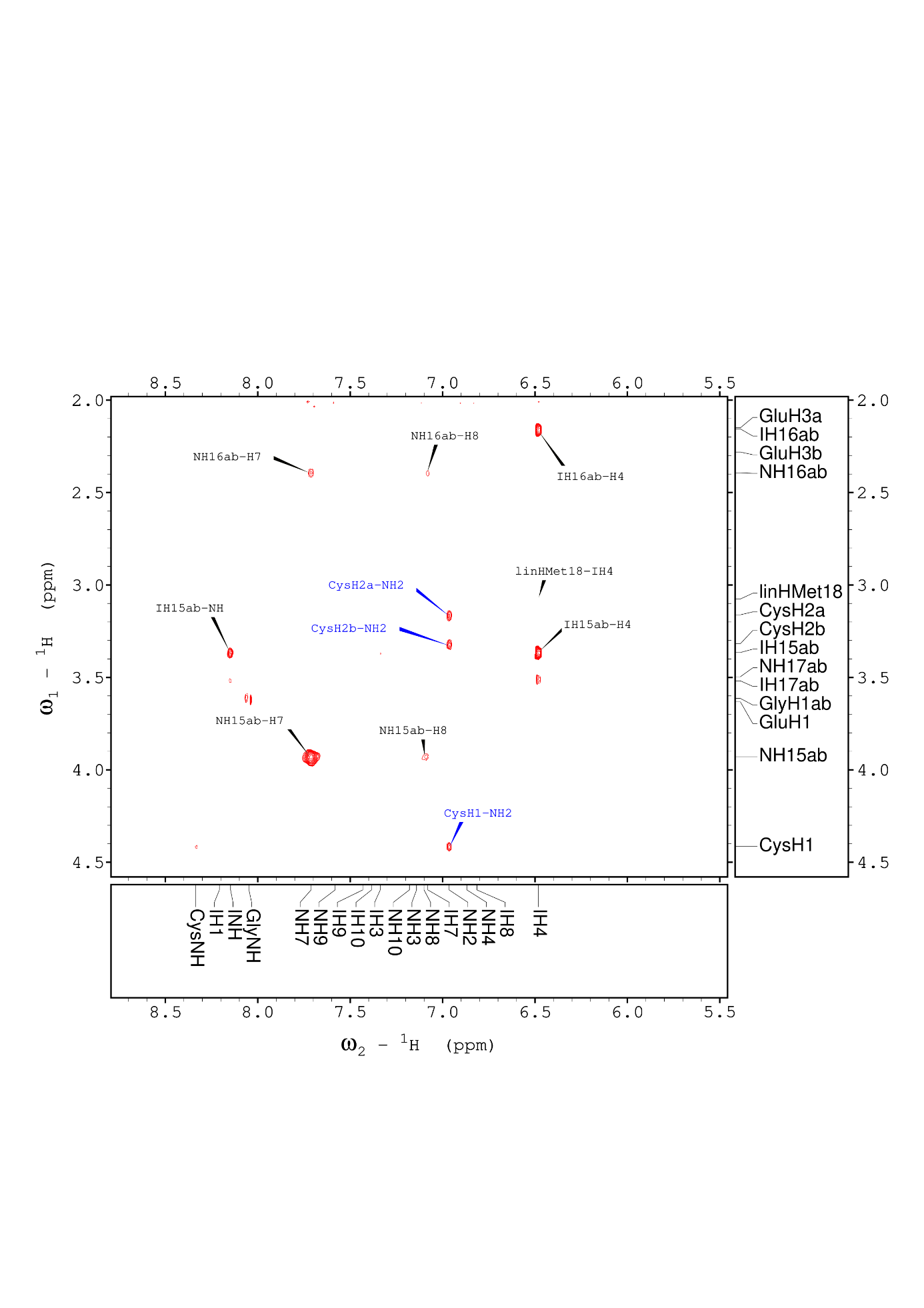
Figure S2.** Fragment of the ^1^H-^1^H NOESY spectrum of the GSH S-conjugate of C-2028, displaying dipolar couplings between the protons of C-2028 and the GSH moiety, marked in blue.

**Table S1.** ^1^H and ^13^C NMR spectral assignment of the GSH S-conjugate of C-2028, based on DQF-COSY, NOESY, HSQC, and HMBC spectra recorded at 55 °C. All the positions are listed as presented in Figure 5. The chemical shifts are reported in (ppm) units using ^1^H residual resonance of water (4.687 ppm) as the internal standard.

| Position | ^1^H chemical shift (ppm) | ^13^C chemical shift (ppm) |
| --- | --- | --- |
| I1 | 8.342 | 136.63 |
| I2 | - | 131.92 |
| I3 | 7.496 | 131.74 |
| I4 | 6.632 | 109.82 |
| I7 | 7.291 | 128.50 |
| I8 | 7.000 | 128.39 |
| I9 | 7.605 | 136.72 |
| I10 | 7.542 | 117.51 |
| I11 | - | 133.61 |
| I12 | - | 104.34 |
| I13 | - | 125.79 |
| I14 | - | 135.75 |
| I15 | **Ha/b:** 3.528 | 41.65 |
| I16 | **Ha/b:** 2.311 | 26.09 |
| I17 | **Ha/b:** 3.699 | 55.30 |
| lin18 | 3.222 | 44.39 |
| N1 | - | 132.29 |
| N2 | 7.156 | 134.56 |
| N3 | 7.346 | 135.86 |
| N4 | 7.097 | 121.45 |
| N5 | 7.848 | 129.01 |
| N6 | 7.247 | 125.69 |
| N7 | 7.732 | 138.25 |
| N8 | 7.358 | 120.34 |
| N11 | - | 113.53 |
| N12 | - | 142.10 |
| N13 | - | 142.14 |
| N14 | - | 113.57 |
| N15 | **Ha/b:** 4.063 | 49.16 |
| N16 | **Ha/b:** 2.521 | 27.62 |
| N17 | **Ha/b:** 3.529 | 54.11 |
| Cys1 | 4.581 | 55.81 |
| Cys2 | **Ha:** 3.327, **Hb:** 3.474 | 40.48 |
| Glu1 | 3.791 | 57.17 |
| Glu2 | **Ha:** 2.050, **Hb:** 2.096 | 29.22 |
| Glu3 | **Ha:** 2.332, **Hb:** 2.444 | 34.47 |
| Gly1 | **Ha/b:** 3.771 | 46.26 |
